# The Influence of Scandium on the Composition and Structure of the Ti-Al Alloy Obtained by “Hydride Technology”

**DOI:** 10.3390/nano11040918

**Published:** 2021-04-03

**Authors:** Natalia Karakchieva, Olga Lepakova, Yuri Abzaev, Victor Sachkov, Irina Kurzina

**Affiliations:** 1 Chemical Technology Laboratory, National Research Tomsk State University, 36 Lenin Avenue, 634050 Tomsk, Russia; itc@spti.tsu.ru (V.S.); kurzina@mail.tsu.ru (I.K.); 2 Tomsk Scientific Center of the Siberian Branch of the Russian Academy of Sciences, 10/4 Akademicheskii Prospekt, 634055 Tomsk, Russia; klavdievna.k@yandex.ru; 3 Material Research Centre for Collective Use, Tomsk State University of Architecture and Building, 2 Solyanaya Square, 634003 Tomsk, Russia; abzaev2010@yandex.ru

**Keywords:** “Hydride Technology”, titanium and aluminum nanopowders, rare earth alloys and compounds, intermetallides, Ti-Al and Ti-Al-Sc systems, lamellar structure

## Abstract

In this study the influence of scandium on the structural and phase state of the Ti-Al alloy obtained by the method of “Hydride Technology” (HT). The Rietveld method has allowed for determining the content of basic phases of the 49at.%Ti-49at.%Al-2at.%Sc system. By means of the methods of transmission electron microscopy (TEM) and X-ray spectral microanalysis, it has been established that scandium additives into the Ti-Al system result in the change of the quantitative content of phases in local regions of the structure. The Ti_2_Al_5_ phase has been found, and Ti_2_Al has been absent. In the morphology of substructures Ti-Al and Ti-Al-Sc there are lamellar structures or lamellae; the peculiarities of the distribution, fraction and size of which are influenced by scandium additives. The average width of Al-rich lamellae has been 0.85 µm, which is four times greater than that for the Ti-Al system (0.21 µm). For Ti-rich lamellae of the sample of the Ti-Al-Sc alloy, the average width of the lamellae has been 0.54 µm, and for Ti-Al it has been 0.34 µm. Based on the obtained data, a scheme of the distribution of phases in the composition of the Ti-Al-Sc alloy in the lamellar structures has been proposed. It has been established that in the Ti-Al-Sc system there is growth of the near-surface strength relative to Ti-Al. In this way, the microhardness of the Ti-Al-Sc alloy has amounted to 1.7 GPa, that is of the Ti-Al alloy which is 1.2 GPa.

## 1. Introduction

Titanium alloys are a sought-after material for creating structural components. They have the necessary properties: they withstand high temperatures, are resistant to corrosion, etc. [1]. Ti-Al-based alloys are heat-resistant alloys for automobile and aircraft industries [2], they are applied in additive manufacture [3]. The morphology of the structure affects the mechanical properties of alloys based on TiAl and Ti_3_Al [4,5,6,7,8,9,10]. The cooling rate affects the growth direction of Al_3_Sc particles [11,12]. During the formation of intermetallides of TiAl or Ti_3_Al, the materials’ strength increases significantly; therefore, their alloying to improve their properties is relevant [13].

To obtain titanium-aluminum alloys, there are many technologies: to identify selective laser melting [14], powder metallurgy [15], laser sintering/melting of aluminum alloy powders [16], physical vapor deposition [17], vacuum arc re-melting [1] and the electric current activated sintering method [18]. The technique of obtaining the Ti-Al-Sc system by the “Hydride Technology” (HT) is of interest for research purposes [19].

Scandium is the major one owing to its increasing applications in aluminum alloys with high strength. China controls the global rare element (RE) distribution by producing over 90% of RE metals. The Global Scandium Market is currently at a nascent stage. There is no primary mine supply of Sc available at present [20]. The high price of Sc (approximately 15,000 USD/kg) limits the extensive commercial application of Sc-containing aluminum alloys. This drives researchers to minimize the concentration of the expensive element (Sc), while maintaining the desirable strengthening provided by precipitation of coherent tri-aluminide L12 nano-dispersoids [21].

Sc-containing alloys have been proven to be attractive materials [22]. Scandium has a significant influence on the structure on the structure and properties of aluminum-based alloys; namely, it affects the formation of grain structure, suppresses recrystallization processes and is a strong hardener of aluminum alloys [1,23,24,25,26]. Scandium, contained in aluminum alloys, improves their physic-chemical properties owing to the fine-grained structure formation [27,28,29,30,31]. Sc serves as a potent grain refiner in castings; scandium additions to the base alloy as well as to welding filler alloys have been shown to have a beneficial influence on weldability and hot cracking resistance of aluminum alloys [27,28].

Sc in the titanium alloy can form the Al_3_Sc phase, which contributes to improving the creep of the titanium alloy [1]. Slight addition of Sc has a positive effect on the yield strength in alloys based on Ti-Al and Ti-48Al having duplex or lamellar structure [5]. Aluminum alloy AA6061 with the addition of 0.15 wt.% can be used in the printing process [3]. Furthermore, scandium is the most effective modifier in aluminum alloys.

Scandium is the most effective element-antirecristallizer in aluminum alloys; Scandium is one of the most effective modifiers of a cast grain structure in aluminum alloys and the strengthening effect brought about by decomposition of scandium solid solution in aluminum decays in semiproducts made from these alloys because of coagulation of these particles during heating processes [32].

## 2. Materials and Methods 

### 2.1. Obtaining Alloys

**Figure 1:**
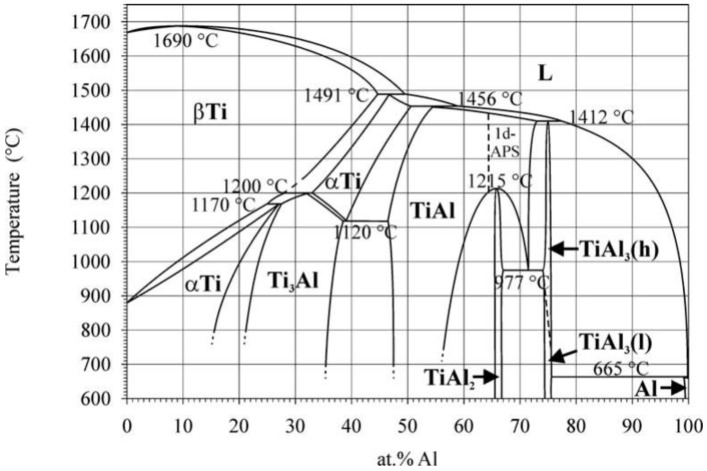
The phase diagram of the Ti-Al system [33].

The samples 50at.%Ti-50at.%Al (Ti-Al) and 49at.%Ti-49at.%Al-2at.%Sc (Ti-Al-Sc) were synthesized by the “Hydride Technology” [19] at a ratio of Ti:Al=1:1 system to obtain an intermetallide γ-TiAl phase (Figure 1) [33]. To obtain samples, titanium (Ti ~96 wt.%; average size—90 nm ± 10 nmAdvanced powder technologies LLC, Tomsk, Russia aluminum powder (loading of active aluminum—81%, particle size—90 ± 10 nm, Advanced powder technologies LLC, Tomsk, Russia) and scandium “SkM-1” (scandium composition ~99.98%, CJSC PRM, Novosibirsk, Russia) were used.

The Ti-Al sample was prepared as follows: a weighed amount of the titanium was heated in tube furnace RSH 120/750/13 (Nabertherm GmbH, Lilienthal, Germany) in a stream of the hydrogen (volume flow of 500 cm^3^/min) to 450 °C. This sample was cured for 3 h at this temperature; after that, it was cooled down to room temperature. The obtained TiH_2_ were mixed with an aluminum powder, pressing under a pressure of 6.63 MPa into a round plate (d = 3 mm).

The Ti-Al-Sc sample was prepared as follows: a weighed amount of the scandium was heated to 550 °C in tube furnace RSH 120/750/13 (Nabertherm GmbH, Lilienthal, Germany) in a hydrogen stream. This sample was cured for 3 h at this temperature; after that, it was cooled down to room temperature. The obtained ScH_x_ were mixed with an aluminum powder, TiH_2_, pressing under a pressure of 6.63 MPa into a round plate d = 3 mm.

The obtained sample Ti-Al and Ti-Al-Sc were obtained in a vacuum (5·10^−6^ atm) unit and heated to a temperature of 1150 °C.

### 2.2. Research Methods

The structural state and the quantitative phase analysis of the system Ti-Al and Ti-Al-Sc samples were studied by the Rietveld method and tranmission electron microscopy (TEM). The X-ray diffraction (XRD) studies of the Ti-Al and Ti-Al-Sc samples were undertaken using DRON4-07 (Bourevestnik, Russia) using copper radiation. The structural state and the quantitative content of the phases were identified by the Rietveld method by means of the reflex [34,35,36]. The crystallographic data of the COD base and the model structures of the Ti-Al and Ti-Al-Sc samples, predicted by the program code USPEX with the interface shell SIESTA, were used as the standard lattices.

The process of manufacturing the samples for the analysis by the TEM method included two stages: cutting on the electrospark discharge machine and preparation of the sample for study on the ion slicer machine (JEOL Ltd., Tokyo, Japan) with an operation mode of U = 7 kW, α = 2 degrees, t = 8 h. The electron microscope studies of the microstructure of Ti-Al and Ti-Al-Sc alloys were conducted using the transmission electron microscope “JEM-2100F” (JEOL Ltd., Tokyo, Japan) using the attachment “JEOL” intended for energy-dispersive spectral analysis (EDS). An accelerating voltage of 200 kV. Phase composition and localization of the formed phases in the samples were investigated by Selected Area Electron Diffraction analysis (SAED patterns).

The microhardness of the alloy samples was measured by the Vickers method, using the microhardness tester PMT-3M (“LOMO“ JSC, St. Petersburg, Russia) by the pressing-in method at an angle with a vertex of 136° under the load of 200 g (Vickers method). In total, 30 indentations were made on the surface of the sample under study.

## 3. Results and Discussion 

Figure 1 shows the phase diagram of Ti-Al [33]. Ti-aluminide alloys have great practical importance in aerospace and automobile industries. Many novel alloys based on γ-TiAl have been developed. The Ti aluminides of industrial importance are mainly based on α2-(Ti_3_Al) and γ-(TiAl) [37].

**Figure 2:**
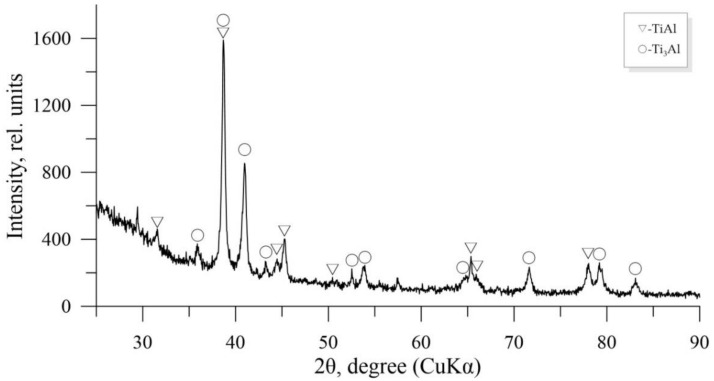
The X-ray diffraction (XRD) pattern of the sintered Ti-Al-Sc sample.

The Rietveld method allows for studying the quantitative content of phases from the integral near-surface regions at the scale level, which includes groups of hundreds and more grains. The results of the X-ray phase analysis of the Ti-Al system obtained by HT showed that basic thermodynamically stable phases included intermetallide compounds of Ti_3_Al, TiAl, TiAl_2_ and a solid solution of aluminum in α-Ti of the variable composition (Figure 2) [19]. The X-ray phase analysis of the samples obtained during alloying with scandium of TiAl-Sc showed that they had a complex multiphase structure [38]. The TiAl phase was a basic phase in the Ti-Al-Sc alloy; its content was 42%. This phase was selected as a base of the alloy and was a matrix. The content of the resulted Ti_3_Al phase amounted to 26%; the amount of the Ti_1.5_Al_2.5_ phase was 11%. In addition, such phrases as Ti_2_Al_5_, Ti_5_Al_11_, TiAl_2_, Al and α-Ti, β-Ti were identified. In this way, the Ti_5_Al_11_ phase formed directly from the reaction of TiAl_3_ and TiAl_2_. 

When comparing the phase composition of the Ti-Al and Ti-Al-Sc systems (Table 1), it is obvious that the addition of scandium in the Ti-Al system changes the quantitative ratio of the resulted phases. In comparison with the initial sample of the Ti-Al system, after addition of Sc, the Ti_2_Al_5_ phase was formed, but the Ti_2_Al phase was not obtained. Apparently, scandium dissolves in intermetallide phases, forming solid solutions, substituting titanium [8]. For both systems Ti-Al and Ti-Al-Sc obtained by the HT method, the phases with a similar type of crystalline lattices were formed. The comparison of the composition of Ti-Al and Ti-Al-Sc alloys, synthesized using HT, and the results of the X-ray phase analysis are given in Table 1.

**Figure 3:**
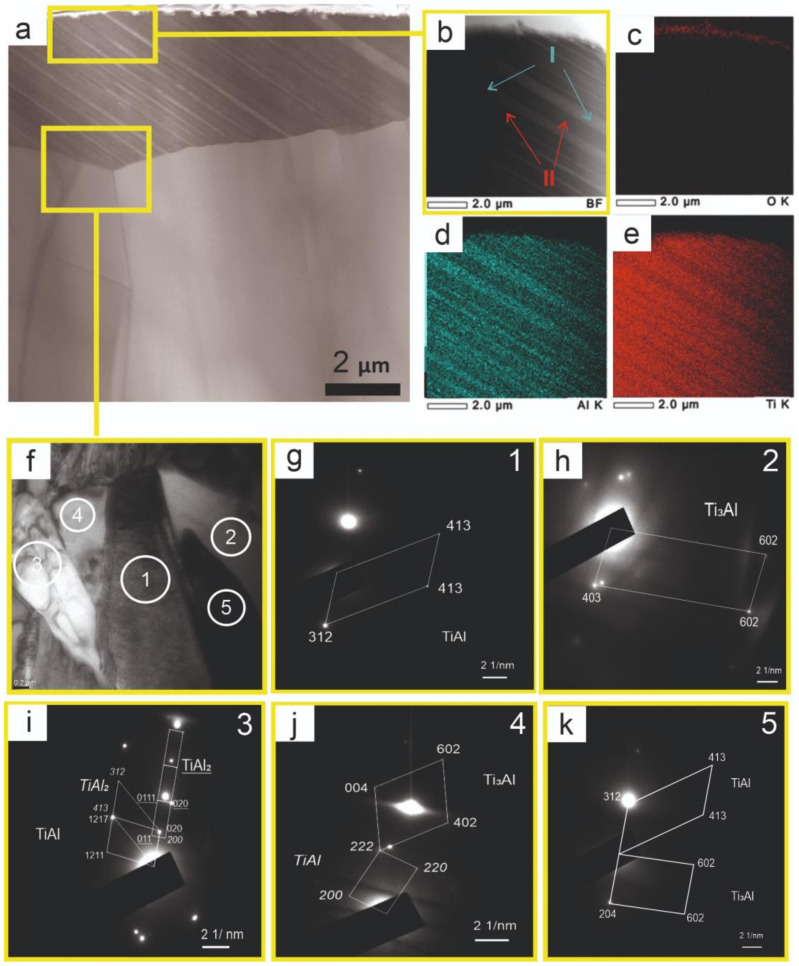
Tranmission electron microscopy (TEM) images of the Ti-Al alloy (**a**,**b**,**f**) with energy-dispersive spectral analysis of the alloys (**c**,**d**,**e**) and the Selected Area Electron Diffraction analysis (SAED) patterns with identification of relevant areas ((1)—**g**, (2)—**h**, (3)—**i**, (4)—**j**, (5)—**k**).

**Figure 4:**
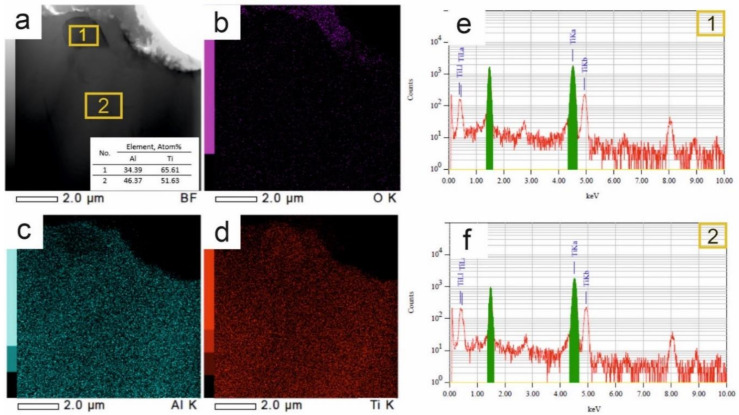
TEM images of the Ti-Al alloy (**a**) with super-spectral surface (**b**–**d**) and with energy-dispersive spectral analysis (EDS) spectrum of relevant areas (1)—**e**, and (2)—**f**.

The materials that are based on titanium and aluminum always have an oxide film on their surface [39]. The obtained oxide film contains a mixture of titanium and aluminum oxides with nonstoichiometric combination Al_x_Ti_y_O mostly in amorphous state. Crystallographic lines of titanium and aluminum oxides are absent on XRD spectra. This work considers the oxide layer that was formed in the Ti-Al (Figure 3a,b). Oxygen is present in small amounts, forming the oxide film only on the sample surface (Figure 3c). The oxide layer thickness on the surface of the Ti-Al alloy is not more than 0.3 µm, which characterizes the low oxidizability of the Ti-Al alloy. The TiAl phase is cubic [39]. Figure 4 shows a TEM image of the Ti-Al alloy in a region close to the samples surface. It is shown that oxygen presented at a surface depth up to 0.5 µm (Figure 4b). According to the elemental analysis (Figure 4c,d), titanium and aluminum are well distributed throughout the volume of the Ti-Al alloy. The elemental composition of this section is confirmed by the energy-dispersive spectral analysis (Figure 4e). 

In the Ti-Al alloy in the homogeneous TiAl matrix, the Ti_2_Al phase is found in the near-surface layer (Figure 4a, zone 1). Titanium and aluminum are observed in an equimolar ratio at a depth of 4 μm and more (Figure 4a,f, zone 2). This results in the formation of the oxide scale on TiAl, leading to the formation of an Al-depleted region beneath the scale [39].

The detailed study of the structural and phase composition of substructures at the scale level of separate grains is possible by means of TEM. When photographing the local regions of foils of the substructure in the Ti-Al alloy, coarse grains of TiAl (of the general type), as well as grains with a layer structure, were found (Figure 3a). In the border areas of grains of the general type, there are particles with various phases (Figure 3f). There are grains with alternating layers, having a fine lamellar structure. The morphology and distribution of ordered domains and their relative orientation in the TiAl phase, coexisting with the TiAl phase in the lamellar structure of Ti-rich TiAl compounds, have been studied by transmission electron microscopy [7]. It has been established that coarse grains belong to the TiAl phase. The analysis of the diffraction patterns has shown that in the first case, intermetallide phases TiAl, TiAl_2_, Ti_3_Al are identified in the near-border regions (Figure 3g–k). Fine lamellae of Al-rich (I type, Figure 3b,d) and Ti-rich (II type, Figure 3b,e)) compounds have been found in grains of the lamellar structure by means of spectral analysis. Ti-rich compounds exhibit better ductility and toughness that the single-phase Al-rich compounds do. It is characteristic of the two-phase Ti-rich TiAl compounds that they exhibit the lamellar structure consisting of the twin-related TiAl and Ti_3_Al phases [7]. It is known that the lamellar structure has a higher crack resistance [37], better plasticity as compared to duplex and solitary γ-TiAl structures. The plasticity of γ-alloys, based on TiAl, with a two-phase lamellar structure depends on the grain size, width of Ti_3_Al lamellae, and the orientation of lamellae with respect to the load axis.

**Figure 5:**
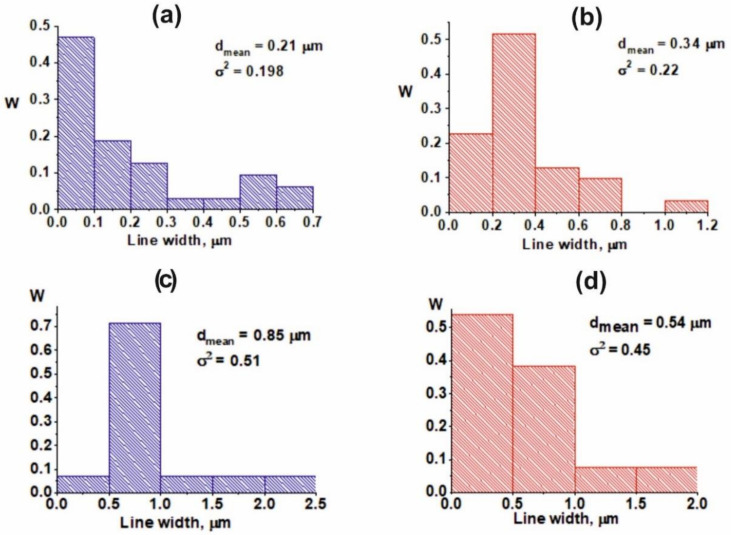
The width distribution of the lamellaes: Ti-Al (**a**—I type, **b**—II type), Ti-Al-2Sc (**c**—I type, **d**—II type).

The width distribution of lamellae of the I type in the Ti-Al alloy is presented in Figure 5a. The average width of the lamellae of the I type has been 0.21 µm. The maximal value of 0.47 of the proportion is typical of the lamellae of the I type up to 0.1 µm wide. The proportion of the lamellae of the I type with a width in the range 0.1–0.2 to 0.19; the proportion of the lamellae of the I type with a width in the range of 0.2–0.3 is 0.13. When considering the width distribution of lamellae of the I type in the range of 0–0.3 µm, the maximum number of lamellae of the I type is characterized by the width, varying from 0.05 to 0.10 µm, and amounts to 0.56. The proportion of lamellae of the I type with a width of 0.1–0.15 µm is 0.16. An identical value of 0.08 has been for the lamellae of the I type with a width in the range of 0.15–0.20, 0.20–2.5 and 0.25–0.30 µm. In the range of 0.40–0.70 µm, the lamellae of the I type with a width of 0.55–0.60 prevail, which amounts to 0.5. The values of the portion of 0.33 and 0.17 correspond to the widths of the lamellae of the I type 0.6–0.65 and 0.47–0.54 µm wide, respectively.

For lamellae of the II type in the Ti-Al alloy, the average width of the lamellae is 0.34 µm (Figure 5b). The maximal value of 0.52 of the proportion is typical of the lamellae of the II type with a width of 0.2–0.4 µm. The proportion of lamellae of the II type with a width, varying within 0 and 0.20, amounts to 0.23; the proportion of lamellae of the II type with a width, ranging within 0.4 and 0.6, is 0.13; 0.6 and 0.8 is 0.1. An insignificant proportion of 0.03 of the lamellae of the II type corresponds to the regions in the range of 1.0–1.2. When considering the width distribution of the lamellae of the II type in the range of 0–0.4 µm, the same number of lamellae is characterized by the width of 0.2–0.25 and 0.35–0.4 µm and amounts to 0.21. The proportion of lamellae of the II type with a width of 0.05–0.1 µm is 0.17. An identical value of 0.125 has been for the range of 0.25–0.35, and the value of the proportion of 0.08 is typical of the lamellae of the II type, which is 0.1–0.2 µm. In the range of the lamellae of the II type with a width of 0.4–1.2, the maximal value of the proportion of 0.57 corresponds to 0.4–0.6 µm. The value of 0.29 corresponds to lamellae of the II type with a width of 0.6–0.8 µm and 0.14 is 1.0–1.2 µm.

The grain substructure in the Ti-Al-Sc alloy has been also studied by the TEM method. In separate grains of Ti-Al-Sc there is a lamellar relief. The width distribution of lamellae in the Ti-Al-Sc system has changed in comparison with the distribution in the Ti-Al system. The lamellae are enriched with atoms of Al (I type) and Ti (II type) (Figure 5c,d). The average width of lamellae of the I type has been 0.85 µm, which is four times greater than that for the Ti-Al system. In this connection, the proportion of lamellae 0.5–1.0 wide of the Ti-Al-Sc alloy is 0.72. A similar value of 0.07 of the widths of lamellae of the I type has been for the ranges of 0–0.5, 1–1.5, 1.5–2 and 2–2.5 µm. For lamellae of the II type of the Ti-Al-Sc alloy sample, the average width of the lamellae has been 0.54 µm. The majority of the lamellae (0.54 of the proportion) are characterized by the width of 0–0.5 µm. The proportion of lamellae of the II type with a width of 0.5–1.0 µm amounts to 0.38. An identical value of 0.07 has been for the range of 1–1.5, 1.5–2 and 2–2.0 µm.

The addition of scandium leads to a modification of the alloy structure. A modification of the primary lamellar structure is observed, which is associated with a change in the boundaries and defragmentation of the lamellar structures [22]. The addition of scandium leads to a change in the elemental composition of the matrix and the volume fraction of γ and α2-phases in the lamellar structural of the alloys. The process of structure modification is related to the grain boundary mobility. In paper [37] it is described the process of growth of lamellar structures by changing the mobility of boundaries and dissolution of primary lamellas and their recrystallization. This mechanism can be implemented in our studies after the introduction of scandium in Ti-Al alloys.

It is known that lamellar structure has a higher creep strength than the classic grain structure of the alloy [22,37]. One explanation for the improved mechanical properties is the peculiarity of the lamellar structure and especially its extended large grain size. The lamellar structure of the alloy allows a significant increase in creep strength above 760 °C. According to [37] the creep resistance of lamellar structure is higher due to the presence of intermetallic α2-phase Ti_3_Al. In our study it is shown that Ti_3_Al phase is formed inside the TiAl-based lamella and acts as a reinforcing component. Intermetallic phases have a special mechanical properties and there present in the alloys can significant increase mechanical characteristics of the alloys.

**Figure 6:**
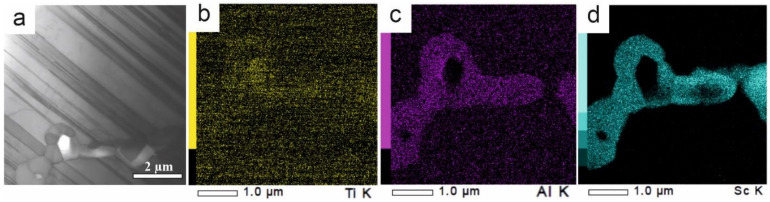
TEM images of the Ti-Al-Sc alloy (**a**) and super-spectral alloy (**b**–**d**).

TEM images of the Ti-Al-Sc alloy are presented in the Figure 6a. A lamellar TiAl structure and isolated scandium inclusions are present. According to the elemental analysis, particles contains titanium (Figure 6b) and predominantly aluminum (Figure 6c) and scandium (Figure 6d).

**Figure 7:**
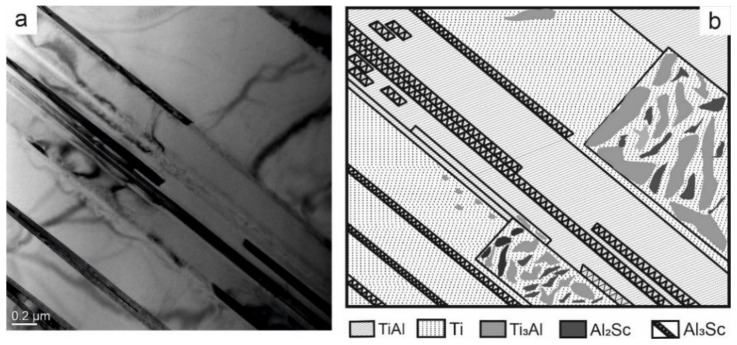
The bright-field image of the Ti-Al-Sc alloy (**a**) and the schema of the phases localization in the Ti-Al-Sc alloy (**b**).

**Figure 8:**
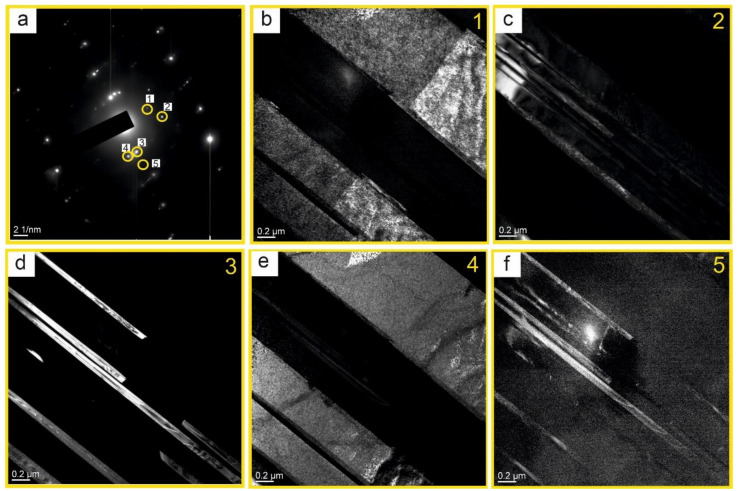
SAED pattern (a) of the Ti-Al-Sc alloy to the bright image, and TEM-dark images in the relevant reflexes (1) —**a**, (2) —**b**, (3) —**c**, (4)—**d**, (5)—**e**, (6)—**f**.

Figure 7a presents a bright-field image of the Ti-Al-Sc alloy with a lamellar structure. There are both wide and narrow layers, differing by the phase composition. To identify them in more detail, the microdiffraction pattern has been interpreted (Figure 8a). The dark-field images have been photographed.

Figure 8a shows the diffraction pattern with dark-field images. Clearly defined lamellae are visible. The TEM has allowed establishing that the matrix of lamellae of the I type is a Ti phase, whose composition contains grains of the Ti_3_Al phase (luminous regions in Figure 8b). For the lamellae of the II type, the matrix is the TiAl, Al_3_Sc phase (luminous regions in Figure 8c). In Figure 8d luminous regions belong to Al_3_Sc, Ti, and TiAl phases (Figure 8f). The grains of the Al_2_Sc phase are between the grains of the Ti_3_Al phase (luminous regions in Figure 8e). To describe visually the results obtained during the interpretation, the layout of the phases has been plotted (Figure 7b). It is significant that when adding scandium, the spreading of the strips is observed. In the matrix of the solid solution of titanium, the formation of the regions with the finely-crystalline structure of intermetallide phases is possible. Intermetallide phases do not form separate strips, but they form separate particles inside or on the basis of alpha-Ti. As a rule, the strips of less thickness are single-phased and alternate with lamellae of the titanium solid solution.

As a rule, the grain substructure with the lamellae structure is free from bend contours. In addition to the lamellar structure in the substructure of the Ti-Al-Sc alloy, there are particles of intermetallide with scandium. The sizes of scandium inclusions are 0.6–0.8 µm.

Scandium additives influence insignificantly the morphology of the grain structure of Ti-Al-Sc. The presence of intermetallide particles of Sc in the Ti-Al system has led to the formation of the stressed state in the particles with surplus contents of Sc. The assessment of internal stresses along the bend contours has been given. The value of the Young’s modulus has been used for the gamma-TiAl 170-GPa alloy [40].

**Figure 9:**
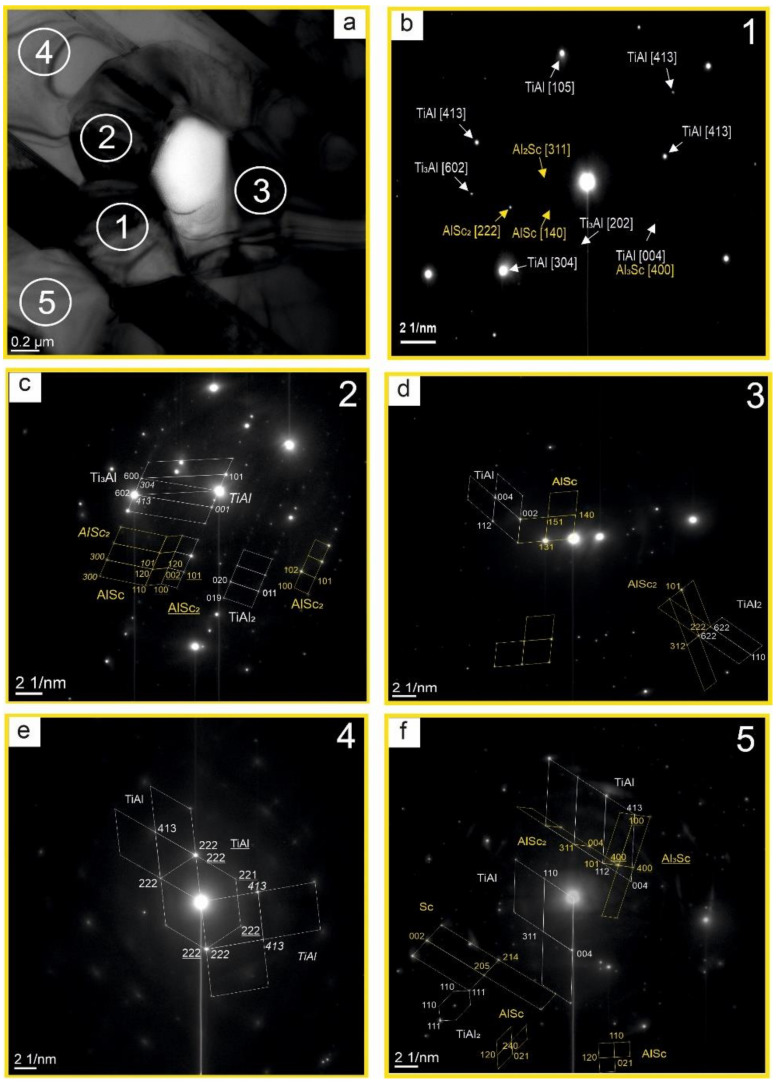
TEM image of the alloy Ti-Al-Sc (**a**) and SAED patterns of the Ti-Al-Sc alloy in the relevant region (1)—**b**, (2)—**c**, (3)—**d**, (4)—**e**, (5)—**f**.

The composition of the particle found in the alloy was investigated by TEM (Figure 9a). Elemental analysis in region 1 showed that a complex multiphase system is formed (Figure 9b). The phases TiAl [413], TiAl [105], TiAl [004], TiAl [304], Ti_3_Al [602], Ti_3_Al [202], and AlSc [140], Al_2_Sc [311], Al_3_Sc [400], AlSc_2_ [222] were found in the sample.

The assessments of internal stresses by the bend contours have shown that for the phases found in point 2 (Figure 9c) the stress in the lattices of the phases TiAl, TiAl_2_, Ti_3_Al, AlSc, AlSc_2_ does not exceed 11.9 GPa. For the TiAl phase, the stress in the lattice of the P4/mmm type in the direction of (001) is 20.4 GPa. For the phases, found in point 3 (Figure 9d), the stress in the lattices of the phases in all directions of TiAl, TiAl_2_, AlSc, AlSc_2_ does not exceed 10.2 GPa. The TiAl phase has been found in point 4 (Figure 9e) in three planes. The stress in the lattices of phases TiAl and *TiAl*, TiAl_2_, Ti_3_Al, AlSc, AlSc_2_ in all directions does not exceed 11.9 GPa. For the TiAl phase, the stress in the lattice of the P4/mmm type in the direction (413 (rhomb, diagonal)) is 27.2 GPa. For the phases, found in point 5, (Figure 9f), the stress in the lattices of phases TiAl, TiAl_2_, AlSc, AlSc_2_, Al_3_Sc, Sc in all directions do not exceed 17 GPa.

**Figure 10:**
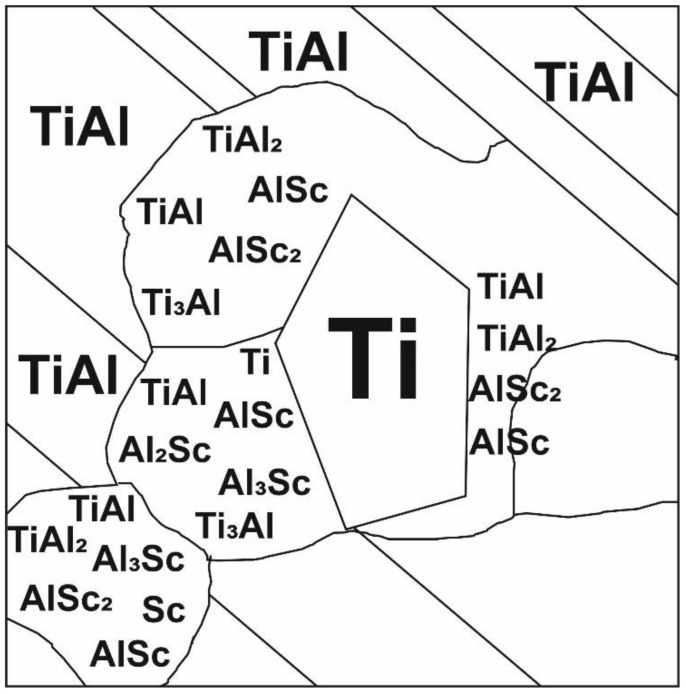
The layout view of the phase distribution on the surface of the Ti-Al-Sc sample obtained by HT.

Based on the obtained results, a distribution pattern of phases Ti, TiAl, TiAl_2_, Ti_3_Al, Sc, AlSc, AlSc_2_, Al_3_Sc in the Ti-Al-Sc alloy is presented (Figure 10). According to the data in Figure 6 and Figure 9, the surface is a matrix of the TiAl composition; a coarse particle represents a scandium agglomerate of the embedded phases. In this way, the center is a titanium phase (luminous regions) (Figure 6). This region is multiphase. All the identified phases are shown in the layout.

In this way, for Ti-Al and Ti-Al-Sc alloys obtained by HT, the microhardness has been studied. For the Ti-Al alloy, the value has been 1.2 GPa, and for the Ti-Al-Sc alloy the value has been 1.7 GPa. An increase in the strength of the alloy with the scandium additive is determined by the decomposition of the supersaturated solid solution, accompanied by the formation of the coherent interface between the matrix and the particle, as a result of which scandium forms the Al_3_Sc phase. This leads to some improvement of strength characteristics, including material creep [1,12,41].

## 4. Conclusions

The possibility of using “Hydride Technology” for production of the new alloys based on Ti-Al and Ti-Al-Sc with lamellar structure is considered. “Hydride Technology” allows us to obtain alloys with lamellar structure and with maximum use of scandium. The formation of the lamellar structures of the fine crystalline regions based on intermetallic phases was observed.

A detailed study of the phase, elemental composition and substructure of Ti-Al and Ti-Al-Sc alloys formed by “Hydride Technology” using transmission microscopy, X-ray diffraction analysis were carried out. The following main phases were found: TiAl, Ti_3_Al and the solid solution of aluminum in α-Ti of variable composition. Scandium has a significant effect on the structure of the Ti-Al alloy. It was found that scandium introduced into Ti-Al included in the new secondary phases: AlSc, AlSc_2_, Al_3_Sc, Al_2_Sc, Sc. It was found that the Sc additions changed the quantitative content of the phases in the Ti-Al alloys.

The study of substructures from local sites by TEM methods revealed two types of grains: grains of general type, as well as lamellar structure type in both Ti-Al and Ti-Al-Sc alloys. The grains of general type contain scandium-containing phases. Moreover, the grains are substantially free of defective substructures both in the body of the grains and in the interfacial regions. Sc additives influence the width of the lamellae as well as the nature of their distribution.

## Figures and Tables

**Table 1:** The phase composition of Ti-Al and Ti-Al-Sc alloys.

Phase.	ΔH° (Formation), kJ/mole [33]	Lattice Type	Ti-Al [19]	Ti-Al-Sc [38]
			Proportion, %	Proportion, %
TiAl	−40.0 ± 1.0	P4/mmm	31	42
Ti_3_Al	−20.3 ± 1.9	P63/mmc	19	26
Ti_1.5_Al_2.5_	-	Pmmm	3	11
Ti_2_Al_5_	-	P4/mmm	3	4
Ti_5_Al_11_	-	I4/mmm	8	4
TiAl_2_	−38.6 ± 2.6	Cmmm	9	3
(TiAl_2_)_1.33_	-	P4/mmm	2	-
Al	-	Fm-3m	1	2
α-Ti	−9.5 ± 1.0	Im-3m	19	6
β-Ti	-	Im-3m	2	2
Total			100	100
